# Journey Through the Fractalization of Multilevel Governance: Levers for Adapt Healthcare Organizations Toward Migrant Populations in Canada

**DOI:** 10.1177/11786329231163006

**Published:** 2023-03-20

**Authors:** Lara Maillet, Paul A Lamarche, Marc Lemire, Bernard Roy

**Affiliations:** 1 Health Administration, ÉNAP, Montréal, QC, Canada; 2 Health Administration, Université de Montréal, Montréal, QC, Canada; 3 Institutional Researcher, Department of Scientific and Technical Development Quality, INSPQ, Montréal, QC, Canada; 4 Nursing Faculty, Université Laval, QC, Canada

**Keywords:** Governance, fractal organization, adaptation, health services, case study, Canada

## Abstract

This article focuses on multilevel governance applied to health organizations in Québec (Canada). The objective is to understand the action levers that facilitate the adaptation of the services toward migrant populations. This type of population establishes itself as an excellent tracer case to analyze the adaptation process, its fractalization and its involvement with the Environment. The dynamics between the actors and their self-organization takes part in the development of a multilevel governance. Interactions with the Environment—both internal and external—highlight the development of networks that emerge from the field and are then implemented at strategic levels in the organizations. The presence of connectivity actors within the organization and the Environment is established. The context, the bonds of trust between the actors and the credibility of the policymakers are reflected as important factors. However, connectivity actors cannot be successful without the support and contribution of the more “hierarchical” actors. Eight action levers are revealed by the analysis. We categorized them in 3 functions: administrative, enabling, and emerging. The levers of the administrative and emerging functions require that the levers of the enabling function be credible and legitimate and be able to support them for the adaptation to spread throughout the healthcare organization, regardless of the scope or policymaking level. The fractal function facilitates this process, by combining connectivity actors with the implementation of connectivity structures.

## Introduction

When it comes to organization theory, stability is traditionally a sign of success and sustainability. This also applies to managers’ vision in health organizations.^1^ However, one can ask whether it is still possible, nowadays, to apprehend the world of organizations—regardless of their domain—as a stable, uniform, and predictable world? It could be the case for some aspects, but in the world of health systems, it goes without saying that technological innovations, socio-demographic changes (aging of the population, migration, low birth rates, work-family life balance, labor shortage, etc.) as well as increasing requirement for flexibility, adaptability and performance do not create the conditions for a traditional approach. Therefore, one of the possibilities for managers, decision-makers and health professionals to give more meaning to their actions is to stay connected to their environment (local context) and to manage the tensions within the institution, service or network. One possible approach for managing these tensions could be, for example, to promote diversity within the organization.^2^

In a previous article, we showed that the interactions between the actors often occur within the same hierarchical level—like through horizontal interactions.^3^ Depending on the context (here: Québec, Canada) and the bonds of trust that may exist between the actors, the proximity between the different levels—like the vertical interactions—is also important when challenges emerge from the field, such as those related to immigration and service adaptation. While primary actors such as nurses and community organizers are *connectivity actors*, they cannot be successful without the support and the contribution of more “hierarchical” actors. To make this happen, the needs emerging from the field must be known and acknowledged.

Through this article, we set out “to identify the action levers, within a multilevel governance, that may or may not facilitate the adaptation of the services to migrants and vulnerable persons.”

For Stroebel et al.,^10^ when primary health care is considered from the perspective of complexity sciences, the focus is more on the quality of the relationships between the actors (inter-professional, professional-patient, administrator-professional, etc.) than on the actors themselves. There exists some sensitivity to the fact that relationships between the various actors are non-linear and dynamical, and consequently, give rise to high levels of surprise and uncertainty. When it comes to knowledge, the preoccupation is based more on continued learning and reflexivity, than on knowledge as an end. It is acknowledged that there exists some interdependency between the formal and the informal organization. It should be noted that it is indeed more important to understand the informal organization than to try (in vain) to underestimate it to the benefit of the sole formal organization. The coevolution between the system and the Environment requires special attention. It is not sufficient to only observe how the system adapts itself to the Environment, one must go beyond and understand the duality of this relation.

The concept of diversity is at the core of the theories of complexity, just like it is at the core of health organizations, with the primary care as the entry door to the system, for most of the population.^4-6^ It is necessary to leverage the diversity that exists between the various actors to promote learning, instead of trying to minimize its effect, or—even worse—to deny it completely.

In our research, diversity refers to the plurality of actors within the health organization (type of occupation, type of vision, values, and culture) but also within the society, particularly in terms of migration. The types of patients targeted by our research are culturally diverse and there is a wide variety of places of origin (homeland, last places of residence, etc.).

Social relationships are also numerous within health organizations; whether binomial (doctor-patient) or collective (team meetings), they promote sense-making, learning, improvisation and other functions that require some interaction between the actors.^7,8^ It is recognized that all managers, professionals, patients, and citizens are parts of the actual system,^9^ and not external observers or manipulators of the system. By considering primary health organizations from this angle, the authors are suggesting that the way they are traditionally seen should be changed from linear entities consisting of a juxtaposition of unique or processual non-related events, to a “*pattern, interrelated processes and relationship”* oriented thinking (p. 440).^10^ This notion of relationships between the actors is shared by other authors who refer to it as *“generative relationship”*.^11^ Indeed, establishing relationships with organizations that represent or provide services to migrant populations and/or newcomers (eg, community organizations) could influence health organizations’ will to adapt their services to this type of patients.^12^ In turn, this will facilitate—within the self-*eco*-organization & co-evolution processes—the overall consideration of this issue by all the actors who play an active part in this process, especially in the governance.^13,14^

After presenting the study’s framework and methodology, the results are split into 3 parts: (1) the levers of administrative functions, (2) the levers of emerging functions, and (3) the levers of enabling functions. The proposal is to regroup all these levers to implicate them in an adaptation framework suited to a fractal organization.

## Study Framework and Methodology

### Framework and research strategy

Leveraging the theoretical framework developed in a previous articles,^15-17^ the model focuses on a complex organization’s multilevel governance.^18-20^ It embeds the determining elements of health organizations in accordance with internal actions (self-organization and feedbacks) and external actions (self-*eco*-organization and coevolution.) This model sets out to situate the characteristics of an organization whose adaptation is fractal, that is, is at all levels of the organization, through connectivity and interdependence mechanisms that support the multilevel governance. Each sphere is subdivided in 3 distinct scopes:

- The **operational** scope represents all of the primary professionals fulfilling a clinical practice, and involved in each case. Under the term “clinical practice,” we include “all clients support processes implying an inherent uncertainty that requires adequate professional judgment”^21^ (p. 170). Clinical activities conducted by primary professionals rely on the degree of adaptation that can be attained in order to sustain and improve the quality, safety, and fairness of the care provided to all patients, including migrants.
- The **tactical** scope refers to actors from the administration and management domains. From a multilevel governance perspective, this scope conveys the strategic orientations to the operational level, and transmits the actions undertaken by the operational level up to the strategic level. By being the junction point between the heterogeneous self-organization of the multiple actors (from the organization and from the Environment), it allows the institution to self-eco-organize itself.
- The **strategic** scope represents the “hierarchical” policymaking entity where the health institution’s senior executives and leaders are situated (General Director, Nursing leadership, etc.). Part of the strategic scope’s responsibility is to bring together and to sustain collective, coherent, unifying and homogenous schemata, while promoting coevolution with the Environment.

Environment actors are the health organization’s external partners. They may include Community Organizations working with migrants (CO), Regional Health Agency, Health and Immigration Ministries, etc.

The selected strategy is a synthetic research through multiple case studies (2 Health and Social Service Centers—(HC))—with integrated analysis levels (3 scopes: strategic, tactical, and operational), in accordance with a qualitative approach.^22^ The choice to focus on 2 Health Centers (HC) in the Monteregian region rather than in Montreal imposed itself, since we were most interested in the adaptation process of those establishments that are “less experienced” in terms of international immigration and cultural mix combined to vulnerability. The study of 2 territories—one urban, one semi-urban—aims to best reflect this reality, consistent with the policies currently implemented in Quebec with regards to immigrant settlement. A regionalization policy being in effect in Quebec,^23^ we set out to better understand how the context can influence health organizations and their willingness to adapt to new phenomena.

The analysis units (the cases) are the 2 HCs (HC1 and HC2) known for being the population’s access points to health and social services—this includes migrant persons.^4,24^

The study focused particularly on 3 clinical programs: family-children-youth (FCY) (Program A), physical health (Program B), and public health & community action (Program C). We selected them because of the importance of the contacts with migrant persons, and the challenges associated. These 3 programs are useful in this research like “observation units” but not like analysis units.

Several administrative services were also retained, in addition to 2 community organizations (CO1, CO2) that are very present in Montérégie region and in the HC territories subjected to the study. Lastly, an actor from the regional branch of the Immigration Ministry (MICC) was interviewed. In total, 43 semi-directive interviews were conducted between November 2010 and February 2011 inclusively. Six (6) final interviews (Immigration ministry, CO1 and 2) were realized in April 2012. We made the deliberate choice to wait for the progression of the analysis of the first 43 interviews, which allowed us to dive deeper on select topics with the actors from the Environment. All interviews were conducted by the same researcher.

#### Eligibility and sampling

Regarding the selection of our respondents, **stratified sampling** was applied to the 2 Health Centers, and **“snowball sampling”** was applied to the actors from the Environment.^25^ Stratified sampling was completed with the help of a key informer in each one of the HC, by selecting respondents within each of the 3 scopes. Additionally, the key informer role allowed us to target—within the 3 programs—persons who worked more frequently with migrant patients and newcomers. This especially applied to the operational scope. However, to limit any selection bias, we also did target stakeholders with less exposure to this type of patients. The objective was to develop a better understanding of the circumstances in which a “less expert” practice, applied to cultural diversity, is experienced by operational actors.

Basing ourselves on Mintzberg’s work (1989),26 we identified 3 scopes: operational, tactical and strategic. The *operational* scope represents all the health professionals who practice in clinics, and who are directly exposed to the patients (nurses, physicians, social workers, and community organizers.) The *tactical* scope represents the managerial part of the organization (lead/supervising-nurse, Human Resource manager, Communication Manager, and Senior Consultants). Finally, the *strategic* scope represents the hierarchical and policymaking area, consisting of directors and chief administrators (general direction, nursing care and professional services directions, clinical direction, members of the board and of tree advisory councils (physicians, nursing, and multidisciplinary).

These 3 scopes are separated between 2 spheres: clinical and administrative. The clinical sphere focuses on phenomena related to the clinical aspects, while managing the interface with the administrative sphere. The administrative sphere focuses on organizational phenomena in interface with the clinical sphere, which it is dedicated to serve.^27^

Actors from the Environment belonged to one of the following 3 types of organizations: (1) 2 community organizations (CO) that were unanimously mentioned by all the HC interviewees, (2) the regional health authority, and (3) the Ministry of Immigration (regional branch). Out of the 57 interviews initially planned, only 8 respondents were not available. It should be noted that it is mostly the respondents from the operational scope of HC1 who refused to participate. In any case, the participation rate for the study was 86%, which is very satisfactory. Frequently, during the interview scheduling phase, respondents would spontaneously get in touch with the researcher, offering to contribute. Actors showed a strong interest for the subject matter addressed by this study.

Data analysis was completed based on the interview transcripts. We conducted a coding analysis by classifying the codes according to the topics and sub-topics obtained through our theoretical framework and through the data itself when the topic was recurrent (Miles and Huberman.^28^ A topic was retained if at least 3 respondents (regardless of their level) mentioned it.

#### Source data collection

Two sources of data were leveraged as part of this research effort: 1- Document sources (N = 21) and 2- individual interviews (n = 49).

1. *Document sources* were used to situate the challenge of migration and health service adaptation at the level of government archives, ministerial archives, and regional agencies, but also at a local level like at the level of HCs and partner community organizations operating and interacting with migrant persons. These documents were minutes of strategic, tactical, or operational meetings such as board meetings, clinical-administrative meetings. These documents were also newspaper articles, minutes of meetings between health organizations and departmental or community partners. Finally, there were also immigration and health policies. This comprehensive documentation search allowed us to enrich the resources provided by the interviewees. For instance, these documentary sources allowed us to better grasp exactly what the Environment is seeking from each one of the studied HC.

*Semi-directive* interviews (N = 49) were conducted with professionals (operational scopes), managers (tactical scopes) and administrators (strategic scopes) (Table 1). For the sampling, we use 2 methods: stratified sampling and “snowball” sampling. Snowball sampling was used for the Environment actors. We did leverage the principle of data saturation in order to stop the data collection.^25,29,30^ An individual interview grid was developed with the objective to cover all the concepts and dimensions from the proposed conceptual framework. Thanks to the flexibility of the interview grid, we were able to tailor it to each group of actors participating in the interviews (operational, tactical, and strategic). We asked the various actors to share ideas or strategies that—from their point of view—could benefit the service adaptation process. To facilitate transcription, interviews were recorded, with the participants’ consent.

**Table 1.** Sample for the study: Interview distribution by site, scope and sphere.

Site	Strategic Scope (n)		Tactical Scope (n)		Operational Scope (n)	Withdrawal (n)	Total
	Administrative sphere	Clinical Sphere	Administrative Sphere	Clinical Sphere	Clinical Sphere		
HC1	4	5	3	3	3	6	18
HC2	4	5	2	2	7	1	20
Regional Agency	4		1			1	5
CO1&2 1 et 2					5		5
MICC			1				1
Total	22		12		15	8	49

Finally, throughout this fieldwork, we kept a *field book* inventorying precise details of the research process (anecdotes, personal thoughts of the researcher, particular events, etc.).^31^ This allowed us to retrace all the activities completed by the researcher, which is a valuable input to the final redaction.

A *“summary sheet”* was completed for each interview.^28^ Each summary sheet was transmitted to the respondents from HC1 so that they could validate and confirm its contents. Because all summary sheets were compliant for all respondents, we postulated that the same approach was not needed with HC 2. HC 1 was selected for practical reasons: interviews were conducted first with HC 1. Moreover, interviews with the Regional Agency and Community Organization 1 were conducted from September to December of 2011. Several respondents from both HCs attended, either in person or through videoconference. The analysis and the preliminary results of the research were presented. Discussions ensued, and the respondent’s expressed satisfaction with regards to the implemented approach. Throughout the study, these restitution checkpoints facilitated information feedback and transparency with the respondents, thus reinforcing the study’s *internal validity* and *credibility*.^25^ Finally, in March 2010, the research protocol successfully met all the criteria set by 2 Ethical committees.

#### Data analysis

The analysis and data gathering activities were performed simultaneously; they included some iterative aspects with regards to coding and categorization, which allowed us to adjust the interview grids. Once the interviews were transcribed, the QDA Miner software—version 3.2^32^—was used to capture the data and facilitate the analysis. Output data were reduced (matrixes, relationship mapping, memos, case summaries), which permitted the development of assumptions that could be verified based on the data already collected, and the data still being collected.^25,28,33^

The analysis was completed in 2 steps: the first step was an *internal case analysis*, by focused on each Health Center. The aim was to regroup and synthesize the models to draw a clear picture of the dynamics and processes within the health center regarding the adaptation of the services to migrant persons.

A deep analysis of each case allowed us to achieve strong internal validity.^22^ Interpretations were verified with actors from different scopes to meet the criterion for credibility (summary sheets for HC1 respondents, and 2 restitutions of the preliminary results involving several respondents from HCs, regional agency, and Immigration ministry).

Moreover, the validation of both the analysis and the interpretations was the subject of several conversations between the research directors and the doctoral candidate.

The second step consisted of a *transverse case analysis*, which allowed us to compare the 2 HCs to expand observations on research proposition. In doing so, we have favored the investigation of explanatory links in order to uncover the mechanisms behind the adaptation process, the interaction between different actors and different levels of governance.^25^

## Results

Basing ourselves on the literary references, we had identified 4 action levers: Structure, Politics, Resources, and *Schemata*.^34^ Four additional ones were highlighted through the coding and interview analysis: “Communication,” “Knowledge,” “Coupling” also known as degree of influence, and “Connectivity.” In the end, 8 action levers are available to test the research proposal (Table 2). Most of them are both favorable and constraining levers to adaptation. Nevertheless, all the respondents agree that they are necessary regardless of their purpose.

**Table 2.** Definitions of the action levers from our conceptual framework (A1) and of the action levers emerging directly from data analysis.

Action lever	Definition
Structure	Role and function of each actor in service adaptation of the services with respect to migrant persons, depending on the established type of management and governance.
Politics	Existence, knowledge and implementation of explicit principles, standards and rules with regards to the adaptation of health services.
Resources	Three types of resources:
	1. *Human* resources: specific human resources that need to be deployed in order to offer adapted services.
	*2. Financial* resources: priority given (or not) to the needs for services related to the treatment of migrant persons, accounting for internal and external constraints.
	*3. Time* resources: this is about the clinical and administrative managers adequately planning for the additional time required from health professionals to self-*eco*-organize in order to assess, orient and follow vulnerable patients, such as some migrant persons.
Schemata	Cognitive structure that determines the action taken by an actor at a given time (t), based on her or his perception of the Environment at time “t minus 1.”
	Individual and heterogeneous characteristic part of the self-organization process. When shared by several actors, the schemata can be collective at department or organization level.
Communication	Information mechanisms put in place to collect/send information from/to all of the actors (indicators, assessments, results) regarding the types of patients serviced, and the different clinical and/or administrative adaptations in effect within the establishments (eg, with respect to patient assessment, orientation and monitoring.)
	Development and promotion of specific means such as a bank of interpreters, or translated and adapted didactic materials, etc. This lever may be utilized in a formal or informal fashion.
Connectivity	Linked established temporally and spatially between the actors of an organization and those of the Environment.^35^ The number of interactions and connections between the actors is more relevant than the strength or weakness of the links themselves: the more links there are, the more they are diverse, and randomly distributed between strong links and weak links. This lever may be utilized in a formal or informal fashion.
Knowledge	Lever leveraging expertise, learning and know-how of the different actors regarding the challenges associated with immigration, and the practice adaptation to such patients.
Coupling	Influence that some actors hold on other actors. This influence is not definite and can vary in intensity over time. It is not necessarily based on a relationship of authority between the actors. Couples are formed, sometime close (*tighted coupled*) and sometime loose (*loosed coupled*)^36^ (pp. 363-364)

*Research proposal: The adaptation of the clinical and administrative spheres and the scopes that compose them operate a convergence through various* ***action levers*** *that facilitate the integration—in a* ***consistent*** *fashion—of the clinical and administrative practices between the professionals, the managers, the administrators, and the Environment.*

In order to analyze these 8 action levers, we have grouped them in 3 overall functions, inspired by the work of Uhl-Bien et al^37^: 1. The « *administrative* » functions—traditional and bureaucratic——are anchored in a vision oriented by control and hierarchical management. They are utilized in a formal fashion. The Structure, Politics and Resources levers are usually found in these functions; 2. The “*emerging*” functions rely on informal dynamics and actions that are under the heels of no authority or hierarchy, thus further fostering creative and learning actions that are based on stakeholders and patients’ needs. Among these functions are usually found the Schemata and *coupling* levers; 3. The “*enabling*” functions allow the organization to adapt optimally, often through problem solving and learning. These functions promote reconciliation between the *administrative* functions and the *emerging* functions. Among these functions are usually found the communication, connectivity, and knowledge levers.

### The action levers in the multilevel governance

#### Levers from the administrative function

The administrative function encompasses—but is not limited to—the formal levers, like the structural, political, and resource-related ones. Following data analysis, 6 main levers were highlighted as belonging primarily to administrative functions.

##### Identifying the official case owner

While *connectivity actors* are mainly found in the operational scope (Community organizers and nurses), it appears that the official designation of a case owner or reference person for the “adaptation & migration” topic is a major thing. Indeed, to everyone, there usually exists a natural interlocutor. It is important that this actor—or this group of actors (such as program C for instance)—have the necessary credibility, through their knowledge of the subject, through their network with the Environment and through their interest for the challenge at stake. Built upon the credibility developed, legitimacy is made possible by the recognition of the role by the hierarchical actors.

*“Like I am saying. . . Having a case owner, let’s say that, for me, around here, for HC2, it is a person you can refer others to. Actually, we know it, it is her who has access to the bank, in case we need interpreters, in case we have an issue with a family, and it’s sort of like that. In order for it to work, it takes a case owner, who is easily accessible, who has relationships with the people that matter in the community, who works with the community”*

[Strategic Actor, Agency]

*“It is necessary to have an owner (for the case), a minimum of resources, and accountability. It also takes willingness; it takes a responsible person. It also takes contracts”*

[Tactical Actor, Clinical, HC2]

As far as Health Center 1 is concerned, the case owner is a collective one. We are talking about programs A and C, via their common program director. However, while the liaisons with the strategic actors are going through him, all the activities—including managing the internal Committee—are delegated to an operational actor recognized as “very much involved” with migrant persons. The “owner” role is shared between the strategic actor and the operational actor, each involved in his own scope of influence. From an internal perspective, as well as from the Environment’s perspective, the operational actor gives credibility to the topic. Meanwhile the strategic actor brings legitimacy via his hierarchical position. In doing so, he facilitates direct access to the clinical and administrative directions.

##### Representation of migrant persons in administrative boards (AB) and users committees

In administrative terms—in addition to the associated symbolism—the demand from cultural and migrant communities to be represented at the Administrative Board is deemed an interesting lever by several respondents. In Health Center 1, this lever is part of the action plan emanating from the internal migration Committee. In Health Center 2, many respondents identified this type of lever as a tangible demonstration of the efforts made by an organization to get adapted and to include people with an immigrant background. However, several stakeholders mentioned that a lot of work is left to be done to bring such an action to completion.

*“Like I said, there could be a reserved spot in the administrative boards of the establishments, for a delegate representing the population, one or two delegates for immigrant populations, why not. So we reserve spots to ensure that they are well represented (. . .) Take the pulse, and have their own weight in the decisions”*

[Strategic Actor, Administrative, HC2]

##### Involvement of the Regional Agency

The involvement of the Regional Agency—specifically on the topic of immigration—is highlighted in several ways. The involvement could, like the Montreal regional bank of interpreters, go through a Monteregian bank relying on what is already existing at the local level, while coordinating the whole thing so that all time slots for accessing these services are expanded. Currently, it is the community organizations that compensate for the lack of interpreters in both the Health Centers studied. At Agency level, the regional vision for the bank of interpreters did not appear to be a priority; this is justified by a weak critical mass of migrant persons and by the fact that local health centers have a responsibility to provide interpreters to patients who speak neither French nor English. However, it is important to mention that several people were mixing up the health centers’ internal banks—which are more informal; the regional bank—which does not exist; and the banks from the local Community Organizations.

*“Yes, I am aware of the bank of interpreters. . . It is organized by the regional agency. . . But I wonder if even internally, we don’t have a small system. . . I am not sure whether this is a formal thing. . . In the field, it is a different story since people know each other, and then, for example. . . if we have a nurse from China. . .., we know it, we leverage her”*

[Strategic Actor, Administrative, HC1]

*“And those, these banks of interpreters are usually managed by community organizations. . . In the Montérégie. This could be different in a region as large as Montreal where some establishments have among their staff people from various communities, who sometimes do offer this kind of services, while doing something else. This is probably not the case in Montérégie”*

[Strategic Actor, Agency]

*“But the establishments that have those communities on their territory, they have the duty to adapt or to do something to reach out to this clientele and to create the conditions for accessible services for those clients. . . And even to help people. . . through interpreters for instance, when they are in need of care or services. And this, this is a preoccupation that must be very. . ., that is very local”*

[Strategic Actor, Agency]

Additionally, the regional Agency had implemented a regional Committee for Health and Social service accessibility by ethno-cultural communities. However, this committee is no longer active for several years. Yet, according to several respondents—including some from the Agency itself—it is the most important lever at regional level.

*“The Regional Committee should be reactivated; this is really important. For the Agency itself, this committee is very important and informs the CEO. The simple fact of formulating, addressing this question with the communities, and developing the* CEO’s *position, this already stimulates the action”*

[Tactical Actor, Regional agency]

Lastly, the leadership of the Agency is directly put in question by some of the respondents. According to them, the Agency’s role is to centralize socio-economical, demographic, and socio-sanitary information to establish—in collaboration with local stakeholders—the primary needs of the populations on those territories. The lack of information regarding migrants and newcomers is raised repeatedly in many interviews.

*“When it comes to program implementation (. . .) there has to be some leadership from the Agency, because part of its role is to better understand the needs of its population and its region, this is why they are here, they are here for consultation, needs (. . .) They have to have some leadership, for me, this is squarely part of their job”*

[Strategic Actor, Administrative, HC2]

*“Within our network, we have very little information regarding the ethno-cultural clientele. We don’t record their background; this is not in the regular databases that we use. (. . .) But at the Agency, we are not aware, and we don’t have it”*

[Tactical Actor, Agency]

##### Development of population-based responsibility and network implementation

The concept of population-based responsibility was highlighted in several interviews. In both Health Centers, program C is especially supporting this challenge inside and outside the organization. The *connectivity actors* attached to this program are trying to educate network actors on this responsibility, while insisting on the importance of sharing this responsibility. By *“Connectivity actors”*, we refer to the actors from the multilevel governance who are involved in the establishment’s adaptation process. Most of time, these actors are from the operational and tactical scopes. In our study, nurses, community organizers and clinical advisors were identified as *“connectivity actors.”* For all of the actors interviewed, regardless of their origin (Health Center, community organization, the Agency or the Ministry), it is clear that the Health Center cannot bear this responsibility alone. Additionally, some actors from the Environment perceive this as an interference from the Health Center, and not as an acknowledgment of their expertise, experience and independence. These tensions are regularly voiced with community organizers, during the MICC’s “Table des Partenaires en Immigration” (Immigration Partners Roundtable meetings) for instance. However, this remains a strong lever. In Health Center 2, it is thanks to the pooling facilitated by the “Table de la Petite Enfance” (Early Childhood Roundtable) that resources were identified, and a liaison officer position created.

*“With the early childhood consultation roundtable, we created a liaison agent position between the families that come in. Very often, they are refugee families with many children (. . .) and it works”*

[Operational Actor, Clinical, HC2]

In Health Center 1, when the topic of immigration comes up, population-based responsibility and network implementation are materialized through the internal Immigration Committee, and through the Health Center’s increased participation in the various adaptation and immigration roundtables and committees. Moreover, this is an interesting lever since it also influences the actors’ schemata versus the network; their ability to share; and the pooling of individual experiences.

*“We live in the same territories, how do we adapt, and in the same time, what are the contributions, the reciprocity, what is available in the community to help us with that, to accompany us and so on. The population-based approach has changed things around, in a way we go back to the early stages of when the LCSC were created: Local Community Service Center. There you go. . . look, there is a difference of vision between: « this is what we have and this guy has to fit in » and « There is this, and us, what are we doing to deliver. . . and to adapt”*

[Strategic Actor, Clinical, HC1]

Combined with population-based responsibility and networks, the development of *reach-out* actions, or of sustainable advanced strategies, is identified as a powerful lever when it comes to service adaptation and vulnerable populations such as some migrants.^38^

*“An organization that reaches out, that is able to go and meet people. . . in their environments, to go. . ., to not simply wait that, that people come ask for help when something is turning, turning bad or. . . It would be proactive to really have, to say “OK, so, where are our migrant populations?”. . . To have, to establish the needs in a more precise way, maybe in our public healthcare plan, well. I think it needs to be clearly defined”*

[Strategic Actor, Clinical, HC1]

##### Increase in the number of employees with immigrant backgrounds, and in the number of “intercultural awareness” employee training sessions

In terms of human resources, the lever most people referred to is the adequacy between the background of the persons employed by the health organization, and the persons treated and followed by the health organization. Without going into systematic *Ethnic Matching*, it is acknowledged by several contributors that the influence of persons with an immigrant background is beneficial to the whole staff, as it facilitates openness as well as the realization that some obstacles do exist, be they cultural, linguistic, or other.

*“I could tell you . . . the story of a. . . lady. . . of Muslim background, anyways, of. . . Muslim confession, who refused to receive care from a male nurse, because according to her religion. . . she could not denude her arm in order for the male nurse to give her the injection. And. . . At this time. . . we had to intervene for, well, well, there wasn’t any, no one else was. . ., no one else was available, so by telling the lady: « Listen, no one else than me or this male nurse is available to provide this service ». At that point she was able to agree to be treated by him. This is just an example. But there. . ., I learned from this example because. . . the nurse, as for him, was upset, his point was. . .: « She shouldn’t have to. . ., well, if she doesn’t want to be cared for by me, then let her go » and all that, and I think this is a good example that shows that we should go beyond the resistance, beyond, and try to understand why, and also adapt our response”*

[Strategic Actor, Clinical, HC1]

In order to reduce the distance between the Environment and the organization, it is important to increase the variability in the organization, in adequacy with the Environment’s variability: Zimmerman and Hurst call that *minimal variability*.^2^

When it comes to resources and knowledge, the lack of training was dispraised by all the interviewed actors. They all agree to put an emphasis on the need for knowledge, be it for operational actors, who interact directly with migrant persons, or for tactical and strategic actors, who need to have at least a basic awareness of the challenges associated with this type of patients.

*“I think it would be good to. . . Could we not. . . It’s difficult to say. . . Go get people from different minorities. Look I know, lately I was asked to go to McGill to attend a recruiting event. There were a couple of people from different backgrounds who were [. . .] (32.10): « send me your resume, I want to do a follow-up ». For sure I want to be certain that they have the right skills, but I said. . . there was a young woman from Arabic background, who speaks English, French and Arabic, and I said she lives in the neighborhood, she knows the area, me I’d like, if she is competent, I’d like her to be part of the team”*

[Strategic Actor, Administrative, HC1]

##### Increase in financial resources allocated to adaptation

The financial resources lever was highlighted in all the interviews as being at the foundation of any possible action. The current budgetary restrictions are limiting service adaptations. Moreover, Bill 100 restrained access to training, which penalizes « long term » learning such as multiethnic trainings.

*“We have zero money for that. We get orders from the ministry: you must offer such and such services. But get organized with the resources you currently have. It is extremely difficult to implement because we just don’t have the resources”*

[Tactical Actor, Clinical, HC2]

*“We are in a context of cost cutting when it comes to training, you have to prioritize. So in the teams, people, they don’t really prioritize because they don’t see it as a need. Because it is not the priority for their clientele. So when you have to prioritize between trainings like. . . For the SIPPE [NDLR: The integrated services and early childhood program] feeding sessions for instance, because there is a need, because if you want to be certified as “Baby-friendly”, we are going to put this as a priority, and not the multiethnic approach”*

[Strategic Actor, Administrative, HC1]

### Levers of the emerging function

The emerging function, as far as it is concerned, regroups the more informal levers, those related to *schemata* and *coupling*. Three main levers were highlighted in the analysis.

#### Involvement of the stakeholders from the environment

Actors from the Environment are seen by the actors from the Health Center as sources of information and immigration experts. In this context, the Health center does not act as a “decision making” entity, but rather as an institutional actor who participates and contributes to a wider network. As the actors from the Environment and those from the Health Center influence each other, a coevolution develops between them. However, Health Centers clearly rely on Community Organizations when it comes to knowledge, networking, information, and population monitoring.

*“CO 2 is the organization, par excellence, that we refer to. We collaborate a lot with them. If at any point we are in need of information, or if we’re not sure about something, we communicate with CO 2”*

[Tactical Actor, Clinical, HC2]

*“For CO 1, the mission is to implement some levers that will facilitate the adaptation of immigrants to their new country. When it comes to health, integrating immigrants in the health care system is not an end in itself but a stepping stone. It is the institution par excellence for integrating all citizens, not only immigrants. This network, at the beginning, must be leveraged for integrating all immigrants, since they are probably excluded from the other networks, especially upon arrival”*

[Actor CO 1]

*“There are things, that are not up to us, be we still have to be able to lead. . . When it’s us, in my opinion, at all times we must be able to offer high quality services, accessibility, continuity in the care, accessibility when it comes to prevention, healing, supporting. This is what we do, community development, we also work with the groups. We do inclusion; we do citizen participation, this is what we do”*

[Strategic Actor, Clinical, HC1]

When it comes to medical practice, meetings between physicians and community organizations prove to be necessary: physicians often complain that they do not know what the available resources are which raises the question of the lack of visibility and *“lobbying”* power from the organizations that support migrant persons on their territory. On the other hand, most of the interviewed nurses working in a Health Center did not indicate that they lacked any knowledge about the resources specialized in migration. On the contrary, they identify them well, know them and most of the time, use them. This highlights the lack of connectivity between the physicians and their Environment as well as the need to join them to get them involved. The monetary aspect was discussed several times, which tends to show that financial arrangements would facilitate greater cooperation from physicians. However, data analysis tends to show that other levers—such as the communication lever—would be just as appropriate. The imbrication of the administrative, emerging, and enabling functions would facilitate physicians’ involvement and would benefit the adaptation of the organization.

On the other hand, a readjustment of the Agency’s Regional Committee would give a “formal” voice to the challenge of adapting the region’s health and social services, and at the same time, permit a contextualization of the actions for each one of the impacted Health Centers.

*“Like I said, activating the Regional Committee is our only hook; there is no other lever available to the Agency. As it stands, it is a structure that was developed by the Agency, not all the Agencies have it, and even Montreal doesn’t have it! The question was posed of whether this service, uh. . . this structure should be abandoned, but in terms of governance, the Agency decided to maintain the advisory committee to the CEO, it’s still something. So we need to leverage this”*

[Tactical Actor, Agency]

#### Factoring operational actors and environment actors’ feedbacks in policymaking

For tactical and strategic actors, considering the feedback provided by operational actors is a way to rapidly gain legitimacy and respect from operational actors. This also facilitates a more patient-oriented care delivery, which proves to be an important thing in the eyes of the operational actors. This type of lever can influence the development of more administrative levers, such as the representation of migrant populations on the establishment’s Administrative Board, for instance.

*“Ideally, this is how it would be done: we all converge towards the same direction, if we are clientele-oriented, we maximize accessibility, we listen to the issues, we identify all that, we find possible solutions, and then we go at it. I think this would be showing true openness”*

[Tactical Actor, Clinical, HC1]

*“When a solution is introduced, it is also evaluated by the whole team (. . .) every six weeks, we meet, and this is how we identify some issues, and this is also how we found some solutions. Indeed, when saying “look at you, you’re from Haiti, this is great what you did that last time”. We give ourselves this way to do things, we hear about these things, from everybody, when we have a client who’s from Haiti, it can be difficult. . . We do it like that, we all work together”*

[Tactical actor, HC1]

As for Health Center 2, it is the needs coming directly from the field that led several actors to develop an informal Committee about service adaptation with migrant patients.

*“Following difficulties that were met, a small committee developed, sort of informally, for service adaptation, and soon we will formalize this approach and gather all the required interlocutors to improve access to services to migrant persons”*

[Tactical actor, Clinical, HC2]

The resonance of the needs and actions from the operational scope permits to create awareness with the tactical and strategic actors on day-to-day clinical operations, and, at the same time, permits to import these challenges in the different scopes so that the willingness to adapt is felt at all levels. It should be noted that in the case of HC2, this process realized itself within the clinical sphere only. Regarding these challenges, the connection with the administrative sphere is more difficult to develop. However, it is acknowledged that some strategic actors would be decisive in terms of *coupling*.

*“I can assure you that identifying the influential actors in the organizations is a big question mark. The financial director has a lot of influence, physicians, money, physicians; all this has a lot, a lot of influence”*

[Strategic Actor, Clinical, HC2]

Their involvement level would then vary according to their schemata. This is not a time for developing and financing innovative programs or actions. This is a time for cost tracking and budget restrictions, and it does not encourage the actors from these departments to get involved in immigration related committees or projects.

*“If we are on a HC territory where there is a strong immigrant population, but no immigrant clients are coming, maybe then we should talk about performance, maybe we are not doing so well. It could be part of our performance indicators, to document for instance the number of immigrants, the number of people coming from another country vs. the number of clients reached by the establishment, and then we could see whether or not we reach vulnerable populations (. . .) Me, I have nothing against performance, I am for performance, I am a manager, the point is to have efficient and reliable services, but efficient and reliable, it also means that we reach the right targets”*

[Strategic actor, clinical, HC2]

### Levers of the enabling function

Each Health Center has levers of the administrative kind, and of the emerging kind. Levers of the enabling function permit the creation of links between the emerging and administrative functions. They gather communication, knowledge and connectivity. Four main levers were highlighted by our analysis.

#### Inter-sphere and inter-scope Communication channels: Breaking the silos via the Board of Communications

The influence of the communication board is a known lever. However, despite the limited means of this board and despite low involvement from strategic actors in this lever, several of the actors interviewed did highlight this specific lever as critical, provided it is implemented adequately. To borrow a Rugby oriented metaphor, just like a try can be converted into additional points, the levers of the emerging functions need to be “converted” by the levers of the enabling function for the adaptation to fractalize and extend through the whole health establishment. Indeed, the winning strategy, according to many stakeholders, leverages the communication lever, be it inter-individuals, inter-program, inter-scope or inter-sphere. It also seems important to break the silos and open the organizations to Environment actors and to the communities. It wasn’t rare for respondents to say that they didn’t *“know those people”* [Operational actor, clinical, HC2] while referring to migrants and newcomers.

*“Since the strategy permits the adaptation, there is no discussion channel (. . .) creating a dialogue between immigrant populations and us, well it can happen through coordination committees, consultation roundtables, but none of it is really. . . Anyways, it doesn’t come to me. The community organizer, well she takes some action, but it doesn’t stick, it doesn’t spread that much through the organization”*

[Strategic Actor, Clinical, HC2]

#### Leveraging “change vehicles” and “connectivity actors.”

Based on public health concerns and community actions, the community organizer can facilitate the transmission of the needs from the field to the strategic actors or to the actors from the administrative sphere.

“I think we can act as a sort of catalyst. We are well positioned because of our community mandates, we know the community well, and we know the organizations well, like [NGO2]. A privileged role in that we can put a name on those realities, and become familiar with them, and bring them to the attention of those other parts of the organization that are less close, and that won’t be confronted to them until a case presents itself”

[Tactical Actor, Clinical, HC2]

*“Community organizers, they can be influential, because they see things, they can say to us: « Hey listen. Think about such and such”*

[Strategic Actor, Administrative, HC1]

However, the community organizer role—by itself—is not sufficient to raise awareness in the organization on the challenges associated with immigration and adaptation. Therefore, the clinical advisor role also becomes an outstanding source of information and knowledge. They make it easier to develop links between the different programs, and they share information with advisors from other establishments. They act as *“connectivity actors”* by directly influencing clinical practice with the operational actors; they also indirectly influence organizational practices by providing information on the needs from the field to the tactical and strategic actors.

*“The specialized care advisors for instance, they are outstanding resources to provide guidance to the teams. Indeed, I am certain that if anything exists, good practices, data, these people can go get that. So I am certain that we are well equipped to address that, and then go fetch what we need to get better tools”*

[Strategic Actor, Administrative, HC1]

“What I say is. . . The advisors, the clinical advisors, they walk around, they (. . .) because the mandate of the advisor is to establish best practices, define what the best practices are. She goes around, she must show openness; consider all avenues to identify the best possibility, and always in relation to the need – at field level, at the level of the clientele. So this is not coming from the top based on what we know, unidirectional, that’s how it is. . .”

[Tactical Actor, Clinical, HC1]

The community organizer and clinical nurse advisor roles have a level of influence that is recognized by all the other actors. They manage to reach all actors, inside and outside of the organization. Later, at policymaking level, any gap will be filled by transmission and relay from the *connectivity* role toward the strategic actors who then may or may not promote this challenge to the various boards.

#### From connectivity actors to connectivity structure

Based on the levers of the emerging function, an informal initiative—carried by one of the community organizers and backed from the beginning by one of the strategic actors—consisted of developing a “Migration” committee within HC1. This initiative was successful, and the committee now involves actors from all 3 fields and both spheres of HC1. Actors from the environment, including community organization 1, also show some involvement. Inter-sphere and inter-scope communication channels materialize, and concrete actions are developed *(Plan for the adaptation of the services to ethno-cultural communities* from Health Center 1); a formal structure is also being implemented, with its official meetings, common objectives and a vision going in the same direction: one consisting of adapting the health organization, at each individual level.

However, it should be recognized that Health Center 1 is not affected by the actions of this Committee, this is especially true for the administrative sphere. On the other hand, each of the actors represented in this Committee becomes a *connectivity actor*, regardless of the scope she or he belongs to. The level of connectivity, combined with the group’s common schemata and combined with the legitimacy of the formal structure that developed from the Committee, promotes the amplification of the challenge throughout the organization and its various scopes: the *connectivity actors* contribute to the development of *connectivity structures*.

*“Me, I used to tell myself, if only I was able to plant a virus within each board, so that they become aware of a global vision, it would make me happy. Even more than that, we are moving towards a work plan. (. . .) The date [of the next meeting] was picked and we are going to hold it at [NGO1] so that we are straight in the right setting, get out of our surroundings, in the end the assessment turned out to be interesting. There is willingness, it’s more than what I thought, I think that the field was maybe. . . Maybe the right timing (. . .) So when we highlighted it, our objective was not to make them a clientele that you have to study with a spyglass, and put aside, but to come to the realization that this is an important part of the population that must be taken into account, so, as leaders and managers, we have some work to do in order to iron out biases, to send a clear sign within the organization that immigration is a priority, then. . .”*

[Operational actor, Clinical, HC1]

Besides, knowledge of existing resources and monitoring trainings are a key action lever. Through efficient inter-level and inter-sphere communication, the knowledge levers allow the affected actors to consolidate their expertise, and in the same time, facilitate continuous improvements in the quality of the care delivered to migrant and newcomer patients. From this point on, structures such as Health Promoting Institutions (HPI) can open themselves to specific certification programs such as the *“Migrant friendly”* program, which favors adaptation.^39^ Clinical and organizational practice adaptation becomes an objective that is shared by all actors—clinical or administrative—via certification procedures that are recognized and valorized: the *connectivity structures* are now established.

*“Let us get certified as « immigration supportive environment » you see? We are « Baby friendly » certified, everyone is trained at different levels: the direct player is going to get a three-day training, the one who is remotely involved in this, the janitor is probably going to get a half-hour session, we don’t have a choice. Everybody, here, we’re all going to be certified”*

[Tactical actor, Clinical, HC1]

*“We are currently implementing the « Health Promoting » concept, in which we can find for instance, the standards related to patient care (and) we could adapt them to Anglophone clientele or clienteles who don’t speak French or English”*

[Strategic Actor, Clinical, HC2]

#### Involvement, commitment, and interest from physicians

It is recognized by several actors that stronger commitment from physicians would be a favorable lever to the adaptation of the organization and to the adaptation of the clinical services. The lack of interest on the part of these actors—together with the shortage of physicians and the peak workloads they are faced with—are some of the factors may explain the low involvement levels.

In this regard, actors from both Health Centers and from the regional Agency did point out again that there exists a paradox between the Health Center’s population-oriented responsibility and the physicians’ professional responsibility. While the Health Center is, by law, accountable for the health and well-being of the population on its territory, physicians—as for them—are accountable for their patients, and they are not required to take more patients that they can or wish to take. According to several respondents, this paradox involves schemata levers and at the same time connectivity and resource levers. The shortage of physicians and health professionals has a negative impact on the levels of involvement with the more vulnerable patients who require more time and knowledge. This knowledge resides directly within the network of partners that physicians may or may not have developed. The more partnerships physicians will entertain with the Environment and with the Health Centers, the more support they will get when delivering treatments and the easier it will for them to access the whole range of services offered. Contrariwise the smaller the network, the more isolated physicians will be in their practice, and they will have to support new types of patients by themselves. This, in turn, will not encourage them to get more involved with this type of patient.

*“It’s just that if you want to raise awareness on a specific clientele, you need to couple that with some support to physicians. When you don’t have that, the door shuts itself. It’s like: « Look, I can’t do any more than this, this is it. ». So, it’s too bad, but that’s the way it is. So, if you want to say to them: « Well, there is such and such clientele that we need to pay special attention to because they have let’s say, one, two, three, four, five, eight particular needs, we need your help », yes, but for such and such things, you need to be more precise, then. . . you will have access to this and that and this and that. Without it, it’s just too heavy”*

[Strategic Actor, Agency]

*“With the labor shortage and the time we. . ., I mean this is the thing we lack the most: time, I don’t think that they are so welcome. We are going to do it if they are sick, but you know, saying: « Yes, I will be your family doctor ». . ., we are not going to rush to say that I think”*

[Strategic Actor, Agency]

The doctor’s symbolic role should also be considered. They hold strong influence and credibility with the actors from the Environment, with the patients, and with the actors from the Health Center. Their commitment in a process like an organization’s adaptation to migrant patients’ treatment gears up the other actors, which facilitates policymaking and action (*coupling.*).

*“It’s all about obtaining commitment from our physicians, from our medical executives. After that it’s up to the president of the CPDP. . . It is a given that the president of the Physicians, Pharmacists and Dentists Council, him, you always have to onboard him from the beginning, this is part of the strategies for sure”*

[Strategic Actor, Administrative, HC1]

As for the enabling function, while physicians’ commitment is a powerful lever, it must be accompanied by administrative type levers, such as easy access to the Health Center stakeholders, for example, the pre-assessment performed by the nurses of the Health Center, which particularly impacts structures and resources.

*“For example, my office is located one kilometer away from the HC; if I had access to a nurse (from the HC) there could be a sort of patient assessment service for migrant populations within the HCs. This should exist, things would be much easier. And physicians would then be more prone to accepting to follow their medical issues”*

[Strategic Actor - Agency]

The paradox opposing population-based responsibility versus the medical or patient responsibility was raised by several respondents; it probably comes from the fact that physicians do not have an obligation to adapt to their Environment. Neither do they have an obligation to get more involved with vulnerable patients like migrants or newcomers. Nevertheless, they are an essential actor of the health system and of its adaptation. In both the cases studied, physicians’ involvement is a key lever, especially in terms of health service accessibility for migrants and newcomers. For example, when a refugee health clinic is created, it is only because there are physicians—in addition to the other health professionals—who agree to see and to follow refugees for a given period.

## Discussion

**The various governance levels, as tools to fractalize the adaptation of the health organization.**

The origin of this article was about identifying the levers of a multilevel governance that may facilitate the adaptation of an organization’s primary health services toward migrant populations. To do so, we set out to verify the following proposal: *“The adaptation of the clinical and administrative spheres of a health organization, and the scopes that compose them operate a convergence through various action levers that facilitate the integration – in a consistent fashion – of the clinical and administrative practices between the professionals, the managers, the administrators and the Environment.”*

Based on the conceptual framework proposed in a previous article, the analysis of the data obtained through a case study focusing on 2 Health Centers and their local, regional, and national Environment allowed us to validate our research proposal.

The different actors, from the 2 Health Centers and from the Environment, leverage several action levers that we grouped in 8 categories (Table 2). To better grasp their dynamics, we classified these 8 levers in 3 functions: administrative, emerging and enabling. It was found that the sought-after consistency between these 3 functions is facilitated by a multilevel governance. The layout and the influence of the types of levers can vary greatly depending on their distribution between the 3 scopes and the 2 spheres. If the administrative and emerging levers benefit from credible and legitimate enabling levers, that are fit to support them, then the adaptation can spread through the health organization, regardless of the scope, sphere, or policymaking level. It spreads in a self-similar or fractal fashion.

**Figure 1.**
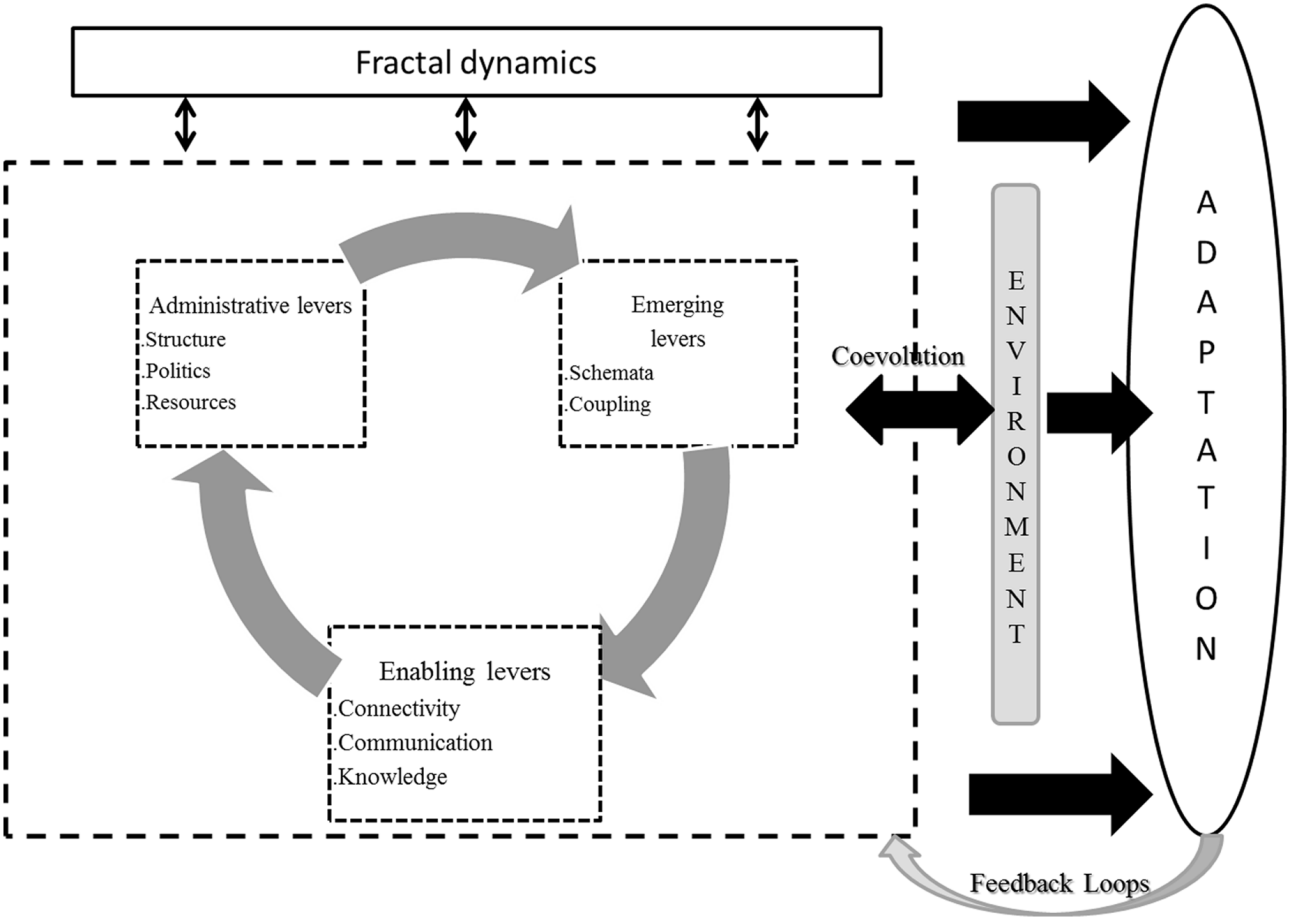
Dynamics between the action levers, the fractalization of an organization’s adaptation, and its Environment.

Figure 1 gives an overview of this dynamics. Although the gearing can be reminiscent of a mechanical system, what we would like to retain here, is the image of the imbrication of the 3 categories of levers. Enabling levers are the largest since the 2 others rely on them. Indeed, administrative, and emerging levers are powerless in the adaptation process, if they are not supported by the enabling levers.

This imbrication allows the fractal function to realize itself through the different levels of an organization’s governance, while coevolving with the Environment actors, first on a local scale then on a global scale. Lastly, the adaptation process is not a “finite” process: over time, retroactions allow for a questioning of the actions that are deemed less appropriate for the programs, for the interventions or for the targeted services. Through these retroactions, it is possible to review the processes, to contextualize them and to learn both individually and collectively.

These 3 functions—that assemble the different levers, themselves paired with the influence of the connectivity actors residing at the different levels of the Health Center—facilitate, even partially, the fractalization of the adaptation within the health organization. It expands over time as actions are taken. The *connectivity actors*, thanks to the information and communication levers, facilitate the development of *connectivity structures*, on which the legitimacy of adaptive actions unequally developed, will then be founded in each scope and each sphere of the organization. The proposal of an adaptation plan, accompanied by structural measures such as representing migrant persons on the user Board of users, can be a demonstration of this fact. This is an enabling lever since it allows the escalation, through the spheres and the scopes, of the needs expressed by the patients or operational actors from the Environment and the Health Center.

The relationships between the Health Centers and the Environment actors play an important part in the adaptation process, and in its fractalization. For example, Health Center 1 relates to several Environment actors, which facilitates the development of partnerships, even more so since HC1’s board was recently replaced. Strategic actors, such as Direction Committees’ members, would like to see HC1 take a more central place within the local health network, since they are eager to operationalize the concept of population-oriented responsibility.

In HC2, the action levers—whether emerging, administrative, or enabling—are not promoted to multiple levels by the *connectivity actors* like in Centre 1. While community organizers and nurses, at the operational level, are recognized as *connectivity actors*, their actions are limited to this level and don’t typically move up to the tactical and strategic levels. Moreover, physicians’ involvement is perceived as weak, yet they represent a major lever especially when it comes to service accessibility. In this configuration, the conditions are not met for the creation of *connectivity structures*. According to some respondents, political and organizational will regarding this challenge is lacking and is too weak to initiate a fractalization of the adaptation. Indeed, it stays at the same scale. Stakeholders’ case-by-case approach and the trial-and-error learning are the most used methods in default of a more homogenous adaptation through all of the scopes and spheres of the governance.

While the traditional hierarchical channels are more present in Health Center 2, the operational actors’ decision-making authority is limited in both the organizations studied. The needs from the field seem more and more remote from the strategic actors and are less and less reflected in their concerns. Tactical actors have trouble bridging the gaps between the 2 other scopes.

However, solutions are being considered when it comes to enabling levers: because it is leveraging *coupling* and *schemata* levers, the communication board could act as an information distributor within the Health Center. It would enjoin the strategic actors—who care about the image of the organization—to get more involved in their organization’s adaptation with migrant and newcomer populations. Nurse advisors, since they reside at the tactical level, are *connectivity actors* capable of creating inter-levels and inter-sphere relationships. They hold a wide array of knowledge as well as a continuously developing network, spreading inside and outside of the Health Center via—among other things—the collaboration they entertain with community organizers. A balance could be developed to facilitate the development of the fractal function and would trigger a multilevel adaptation process comprised of continuous support between the different actors and establishments.

The fractalization of an organization’s adaptation allows the necessary adjustments to be achieved in a coordinated fashion, while avoiding redundancy between the different scopes and the different spheres. Because the clinical governance tends to leverage the emerging levers while the administrative governance tends to leverage the administrative ones, a junction point is possible, provided the enabling function is applied to the multilevel property of the organization’s governance. If not, there is a significant risk to become stagnant within one scope without answer for wicked problem or global crisis.^40,41^ This does not permit more than a heterogeneous, case-by-case adaptation, with limited individual and collective learning.

To Fractalize a health organization’s adaptation is to bring together several necessary characteristics: (1) *connectivity actors* in the different scopes of the organization; (2) administrative and emerging levers exist and are relayed by enabling levers, which allows them to be tied together to realize concrete actions at different levels within the organization; (3) a network-based organization interacting with diverse Environment actors. However, a unique actor-partner like in the case of Health Center 2 presents a risk of monopolizing the attention and the tension between this organization and the operational actors, to the detriment of the recognition of the challenge, and of a hierarchical and organizational support.^42^ (4) Lastly, support from the Environment actors at the regional and national levels appears to be important to facilitate the globalization of the challenge as well as a more systemic approach.

In a nutshell, the adaptation of a health organization is « fractalized » through the implementation of action levers that make it more visible and present at each governance level, thanks to *connectivity structures*, who are themselves facilitated by the presence and the involvement of *connectivity actors*. This pleads for a new approach to adaptation processes, where priority would be given first to the actors, and second to the structures.

### Study limitations and future research directions

We address here the limits. There are 3 of them. The first concerns the choice we had to make for reasons of feasibility. Indeed, in addition to obtaining the point of view of the actors of the health organization and the environment, we would have liked to collect the point of view of migrant people who had or had not had access to health services. However, we were careful to take into account the literature and the reports of community organizations so as not to exclude more community than institutional points of view.

The second limitation concerns internal validity: the views of some professionals may have been “diminished” due to less participation in interviews.

The third limitation is that our case study was conducted, like many research studies, over a 4-year period (2010-2014). This allows for the collection of actors’ positions at a given time, in addition to historically tracing the different stages of the organization via their memories and the administrative documents consulted. However, since our fieldwork, several major transformations have taken place, such as a major reform of the Quebec health system in 2015.

At the end of this article, we wish to highlight 3 major points arising from our results:

The first concerns a point raised by many of the actors we met: the lack of visibility of migrant patients (and their representatives) within the governing bodies of health organizations such as boards of directors (BoDs) and user councils.

The second concerns the politicization of issues related to immigration and the integration of people of immigrant background into the host society. This affects the health care environment and can play a negative role for those with real needs, whether they are migrants or not. This contributes to the emphasis on the health care system as an “integrating” system for “non-integrated” populations, posing “problems” because it does not respond to the law of the majority. This type of reasoning and vision frequently serves parts of the population that do not have the capital, at the appropriate time, to take their place in the social debate. Thus, in the perspective of a just and equitable society, and without talking about depoliticizing the health system, it would be important to maintain, at all costs, a “safety net” for people in precarious and vulnerable situations, whether economic, social, or material. In terms of migration, for example, mental health services, especially for “refugee” migrants, pose major problems (and questions): indeed, their care is insufficient, with many breaks in follow-up, and even refusals of care.

The third concerns the context favorable to the establishment and emergence of networks: indeed, a tendency toward adapted governance in networks, like more balanced to respond to the ambiguity of adaptation in complex organizations, has been revealed. Indeed, within the 2 health organizations studied, and their respective environments, we were able to observe on several occasions that the concept of population responsibility is increasingly integrated by the different actors.

There is therefore a dynamic that calls for a strengthening of the concept of population responsibility, while pluralizing it to all the actors involved, and not simply defining it as the preserve of the targeted health centers. These types of networks, as we have seen, go beyond the health system alone and therefore cannot be limited to typically “health” coordination.

## Conclusion

The presence of connectivity actors within the organization and the environment is revealed. The context, the bonds of trust forged between actors and the credibility of decision-makers appear to be important levers.

But the connectivity actors cannot achieve this without the support and contribution of the more “hierarchical” actors. Eight levers for action emerged from the analysis. We have classified them into 3 functions: administrative, enabling, and emergent.

Through the “connectivity actors” emerge the needs of the field. If they are heard and understood by the strategic actors, the structures in turn become “connectivity structures.

The levers of the administrative and emerging functions require that the levers of the enabling function be credible and legitimate and be able to support them for the adaptation to spread throughout the healthcare organization, regardless of the scope or policymaking level. The fractal function facilitates this process, by combining connectivity actors with the implementation of connectivity structures.
